# Seasonal and spatial variability in rates of primary production and detritus release by intertidal stands of *Laminaria digitata* and *Saccharina latissima* on wave‐exposed shores in the northeast Atlantic

**DOI:** 10.1002/ece3.10146

**Published:** 2023-06-20

**Authors:** Abby R. Gilson, Lydia J. White, Michael T. Burrows, Dan A. Smale, Nessa E. O'Connor

**Affiliations:** ^1^ School of Biological Sciences, Institute of Global Food Security Queen's University Belfast Belfast UK; ^2^ Tvärminne Zoological Station University of Helsinki Hanko Finland; ^3^ Scottish Association for Marine Science Scottish Marine Institute Oban UK; ^4^ Marine Biological Association of the UK Plymouth UK; ^5^ Present address: Trinity College Dublin, School of Natural Sciences Trinity College Dublin Dublin 2 Ireland

**Keywords:** carbon cycle, detrital production, ecosystem functioning, *Laminaria digitata*, macroalgae, primary productivity, *Saccharina latissima*, temperate reefs

## Abstract

Coastal habitats are increasingly recognized as fundamentally important components of global carbon cycles, but the rates of carbon flow associated with marine macrophytes are not well resolved for many species in many regions. We quantified density, rates of primary productivity, and detritus production of intertidal stands of two common intertidal kelp species—*Laminaria digitata* (oarweed) and *Saccharina latissima* (sugar kelp)—on four NE Atlantic rocky shores over 22 months. The density of *L. digitata* was greater at exposed compared to moderately exposed shores but remained consistently low for *S. latissima* throughout the survey period. Individual productivity and erosion rates of *L. digitata* did not differ between exposed and moderately exposed shores but differed across exposure levels throughout the year at moderately exposed sites only. Productivity and erosion of *S. latissima* remained low on moderately exposed shores and showed no clear seasonal pattern. Patterns of productivity and total detrital production (erosion and dislodgement) per m^2^ of both *L. digitata* and *S. latissima* followed closely that of densities per m^2^, peaking in May during both survey years. Temperature and light were key factors affecting the productivity rates of *L. digitata* and *S. latissima*. Erosion rates of *L. digitata* were affected by wave exposure, temperature, light, grazing, and epiphyte cover, but only temperature‐affected erosion of *S. latissima*. Production of biomass and detritus was greater in *L. digitata* than in *S. latissima* and exceeded previous estimates for subtidal and warmer‐water affinity kelp populations (e.g., *Laminaria ochroleuca*). These biogenic habitats are clearly important contributors to the coastal carbon cycle that have been overlooked previously and should be included in future ecosystem models. Further work is required to determine the areal extent of kelp stands in intertidal and shallow subtidal habitats, which is needed to scale up local production estimates to entire coastlines.

## Graphical abstract

Using long‐term field surveys, we present new data quantifying key processes (production and detrital production) in an intertidal kelp system under differing environmental conditions. We show that density and productivity of intertidal kelp differ between kelp species and levels of wave exposure and, as predicted, that rates of productivity and erosion vary seasonally. We also identify temperature, light, and wave exposure as primary factors affecting these key processes, along with grazing pressure and epiphyte cover. Finally, we show that per m^2^, production and detrital production of intertidal kelps is comparable to that of subtidal and range‐expanding warm‐temperate species, highlighting the important contribution of these habitats to the coastal carbon cycle that has been previously overlooked.

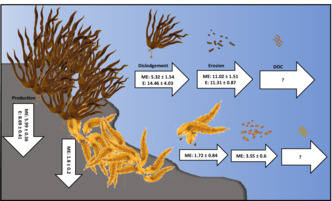

## 1 INTRODUCTION

Coastal vegetative habitats (e.g., mangrove forests, salt marshes, seagrass meadows) have long been recognized as important carbon sinks (i.e., blue carbon) owing to extremely high rates of productivity and capacity for local carbon storage (Bauer et al., 2013; Duarte, 2017; Duarte et al., 2005). Increasingly, macroalgal habitats (i.e. fucoid and kelp forests) are included in the blue carbon conversation due to their extremely high productivity and spatial extent (Pessarrodona et al., 2022) even though they do not store carbon locally within sediments. Carbon flows through these coastal ecosystems via multiple trophic pathways, many of which play a fundamental role in regulating rates of ecosystem functioning (Byrnes et al., 2011; Steneck et al., 2002). These pathways, however, remain unresolved in many systems and the mechanisms by which carbon flows through different compartments of the coastal carbon cycle are understood poorly.

Macroalgal habitats represent the most productive and extensive of the coastal vegetative habitats (Duarte, 2017; Duarte et al., 2022), with maximum productivity estimates exceeding ~1000 g C m^−2^ year^−1^ in the North Atlantic (Mann, 1973, 2009) and ~5000 g C m^−2^ year^−1^ globally (Pessarrodona et al., 2022). It is estimated that intertidal and subtidal macrophytes may contribute up to 45% of total primary production in some near‐coastal systems (Smale et al., 2013). Most of this production comes from large brown seaweeds (e.g., kelps and fucoids), which form extensive stands, primarily along temperate and polar rocky coastlines (Duarte et al., 2022; Steneck et al., 2002). These habitats are characterized by extremely high rates of carbon fixation, supporting high secondary production and creating biodiversity hotspots that support many commercially important species (Smale et al., 2013). Kelp productivity correlates strongly with a number of environmental variables, including nutrients, light, temperature, and wave exposure (de Bettignies et al., 2013; Graham et al., 2007; Hurd, 2000; Krumhansl & Scheibling, 2012; Smale et al., 2016, 2020). This sensitivity to environmental factors has resulted in significant changes to productivity and biomass, with the potential to have large indirect effects on coastal food webs and ultimately ecosystem functioning and stability under future environmental change scenarios (Wernberg et al., 2019).

The majority of kelp‐derived production (>80%) enters the food web through detrital pathways, with high rates of export from source populations and the potential for long‐distance transport to recipient ecosystems (Krumhansl & Scheibling, 2012). This transfer of carbon has been shown to constitute a crucial trophic subsidy in a range of habitats, including rocky shores, sandy beaches, submarine canyons, and the deep‐sea (Gilson, Smale, Burrows, et al., 2021; Krumhansl & Scheibling, 2012; Polis et al., 1997). Detrital production is generated by two primary mechanisms, chronic erosion of material (typically from the distal part of the blade) or dislodgment of sections or entire thalli (de Bettignies et al., 2013; Krumhansl & Scheibling, 2011). Depending on the mechanism detrital properties, such as particle size and density, can vary and influence rates of transport and consumption (Filbee‐Dexter et al., 2018). Wave action is often considered to be the primary driver of kelp detritus production, owing to the accumulation of wrack in coastal habitats after storms and the higher rates of removal observed during storms, particularly for whole thalli (Dayton & Tegner, 1984; Milligan & DeWreede, 2000; Seymour et al., 1989). Temperature, however, has been positively correlated with erosion rates, with higher erosion rates typically occurring during summer and autumn months (de Bettignies et al., 2013; Hereward et al., 2018; Krumhansl & Scheibling, 2011). Biological factors, such as epiphyte cover, grazing pressure, and kelp fecundity, have also been linked to erosion rates through the structural weakening of kelp tissue (de Bettignies et al., 2013).

Although data remain relatively limited, a recent surge in research efforts has yielded important insights into primary production and detritus release in kelp forests (Dolliver & O'Connor, 2022). Despite this, studies are largely restricted to a few geographical areas, particularly Australasia and North America, with comparatively fewer in Europe, including Ireland and the UK (Smale et al., 2013). In recent years, work in the UK has begun to characterize kelp forest structure using systematic large‐scale field surveys, quantifying the density and distribution of subtidal kelp forests and linking regional‐scale variability with environmental variables (Hereward et al., 2018; Pessarrodona, Foggo, et al., 2018; Pessarrodona, Moore, et al., 2018; Smale et al., 2016, 2020; Smale & Moore, 2017; Smith et al., 2022).

Few studies have quantified primary production or detrital release by intertidal kelp stands, despite clear differences in environmental conditions, community composition, functional traits, and food web structure between intertidal and subtidal habitats (Hereward et al., 2018). For example, out of >1000 global estimates of macroalgal primary productivity, only 37% are intertidal estimates and <2% are intertidal kelps (Pessarrodona et al., 2021). Unlike subtidal habitats, the intertidal zone is influenced by both oceanic and atmospheric climates and experiences a steep stress gradient associated with tidal cycles. It is expected, therefore, to exhibit a pronounced response to climate change impacts that may differ significantly from those seen in subtidal habitats (Hawkins et al., 2009; Helmuth et al., 2006). Although intertidal kelp stands are restricted to the very low shore fringe and cover a much smaller area than subtidal stands (Yesson et al., 2015), dominant species can occur in greater densities, suggesting that per area unit they may make significant contributions to coastal primary productivity. Reliable estimates of carbon fixation and fluxes are lacking for wave‐exposed extreme‐low shore habitats in most regions, however, most likely because of their inaccessibility.

Having identified these knowledge gaps, we estimated rates of primary production and detritus release by intertidal stands of two kelp species widely distributed across the North Atlantic. We examined seasonality and the influence of wave exposure on carbon dynamics and tested whether biotic (grazing pressure, epiphyte algal cover) and abiotic (temperature, light) factors affected kelp production and breakdown on wave‐exposed rocky shores in the northeast Atlantic.

## 2 METHODS

### 2.1 Study design and location

**FIGURE 1:**
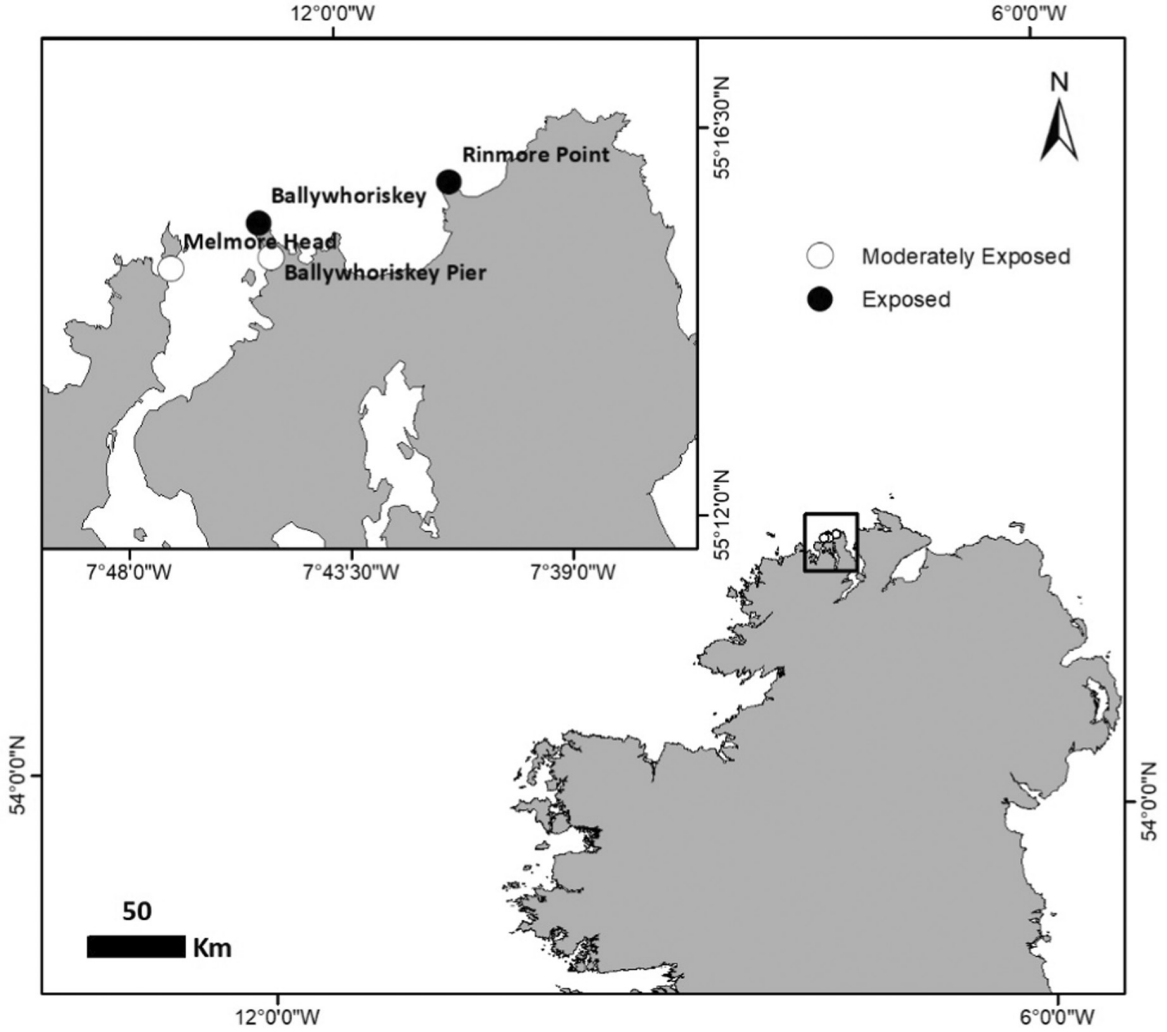
Study sites were at exposed (Ballywhoriskey Point and Rinmore Point) and moderately exposed (Ballywhoriskey Pier and Melmore Head) shores in Co. Donegal, Ireland.

We quantified density, productivity, erosion, and dislodgement of intertidal stands of *Laminaria digitata* and *Saccharina latissima* seasonally over 2 years (in May, August, and November 2016; February, May, August, and November 2017; February 2018). For *L. digitata*, we tested for the effects of wave exposure by quantifying these aspects of kelp populations at two exposed (Ballywhoriskey and Rinmore Point) and two moderately exposed (Ballywhoriskey Pier and Melmore Head) sites (Figure 1). We quantified carbon dynamics for *S. latissima* only at the two moderately exposed sites where it occurred (Figure 1). We also quantified grazer abundance and damage, epiphytic algal cover, temperature, and light levels as potentially important in influencing the observed patterns. To test for anticipated seasonal responses, sampling dates were chosen to reflect spring, summer, autumn, and winter. Some sampling dates, however, do not fall distinctly within meteorological seasons owing to the 4 week‐period between tagging individuals and data collection. We, therefore, refer to them as sampling periods instead of seasons.

**FIGURE A1:**
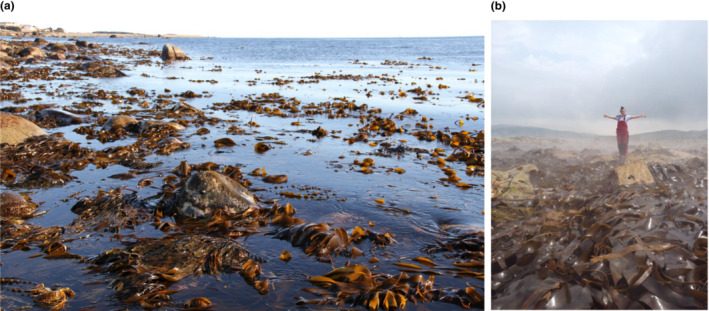
*Laminaria digitata* and *Sacharina latissima* kelp beds located at (a) Ballywhoriskey Point and (b) Rinmore Point in Co. Donegal, Ireland.

Sites were located on the NW coast of Ireland in Co. Donegal and are typical of open coast shores in the wider NE Atlantic region (Mrowicki et al., 2014; O'Connor et al., 2011). Sites were selected based on their simulated average wave fetch (*F*) from a vector‐based digital coastline model (Ballywhoriskey 5415.9 m, Rinmore Point 5460.1 m, Ballywhoriskey Pier 1224.8 m and Melmore Head 1224.9 m; Figure 1; Burrows, 2012). All sites were characterized by large gently sloping granite platforms that were characterized by a patchwork of barnacles and juvenile mussel beds (particularly at exposed sites), and dense macroalgal canopies interspersed with patches of bare rock. On moderately exposed shores, a band of *S. latissima* extends below the fucoid region, before giving way to *L. digitata* beds at the extremely low intertidal zone (1.0–1.5 m above Chart Datum; Figure A1). On exposed shores, *L. digitata* dominates the low shore and sparse stands of *Alaria esculenta* occur attached to large boulders located within the kelp beds (0.86 ± 0.2 individuals per m^2^). On all shores, individuals of large brown macroalga *Sacchoriza polyschides* are interspersed sporadically among the dominant kelp species (0.16 ± 0.03 individuals per m^2^ based on quadrat surveys described below).

To quantify the density of both kelp species at each site during each sampling period, stratified haphazard sampling was used to place between 8 and 10 quadrats (0.25 m^2^) on bedrock within the kelp bed habitat (0.3–0.8 m above chart datum). The density of mature *L. digitata* and *S. latissima* individuals (i.e. canopy formers) was recorded in each quadrat.

To estimate the productivity rates of *L. digitata* and *S. latissima* during each sampling period, 15–20 mature canopy‐forming individuals (>1 m) of each species were selected randomly at each site and tagged individually. Juvenile kelps were excluded from the current study owing to their representation of only a small proportion of these kelp populations and time constraints. In addition, juvenile recruits are spatially patchy and constrained by different environmental variables. Elongation rates and biomass accumulation of each individual were estimated using a modified hole‐punch method (Tala & Edding, 2005). Some individuals were lost due to wave dislodgement such that final sample sizes varied from 3 to 17 individuals of each species per site per sampling period. For *S. latissima*, each individual was punched with one hole located 10 cm from the stipe/lamina junction. For *L. digitata*, because it forms a digitated blade, three holes were punched, the first and second 10 and 20 cm above the base of the central lamina, respectively, and the third 10 cm above the base of the blade on the first digit. After 4 weeks, tagged individuals were relocated and growth was measured. For *S. latissima*, the distance between the first hole and the base of the blade and the final blade length were measured. The growth rate was then calculated as:

$$G=\left(\mathrm{Hf}-10\right)/t,$$

where Hf is the final growth hole position (cm) and *t* is the number of days between initial and final measurements (Tala & Edding, 2005). For *L. digitata*, the distance of all three holes from the base of the blade was measured and growth rate was then calculated using the mean of the three measurements.

Productivity was calculated for each species as the average estimated dry biomass per unit length for the basal 1/3rd of the thallus multiplied by the growth rate (g DW day^−1^). Dry biomass per unit length was estimated by taking 5 cm sections of the stipe, basal, and distal 1/3rd of the blade, and obtaining the wet weight before drying in an oven at 60°C until constant weight. A relationship between wet and dry biomass (g cm^−1^) was then established for the stipe, basal, and distal 1/3rd of the blade using linear regression (*p* ≤ .05; *R* ^2^ > .80).

Rates of detrital production in *S. latissima*, were estimated from tissue loss from the thallus (TL, cm) based on the change in blade length and blade growth:

$$\mathrm{TL}=\left(\mathrm{BLi}+g\right)-\mathrm{BLf},$$

where BLi and BLf are initial and final blade length (cm) and *g* is the length of the new tissue produced (cm). For *L. digitata*, the same equation was used for both the center and outer digit and an average taken. The rate of erosion (g DW day^−1^) was then calculated as the average estimated dry biomass per unit length for the distal 1/3rd of the blade multiplied by the tissue loss and divided by the number of days between sampling occasions.

To estimate kelp dislodgement rates, the 15–20 individuals tagged previously were collected and dislodgement was assumed from missing tagged individuals. Dislodgement rate (% dislodgement per day) was then defined as the difference between the initial and final number of tagged individuals between sampling periods divided by the initial number. Dry biomass loss through dislodgement was then estimated using the relationship between wet and dry biomass for the whole individual. Owing to adverse weather conditions, data were not available for August and November 2017.

To estimate daily productivity and erosion rates per unit area, individual productivity and erosion rates for *L. digitata* and *S. latissima* were multiplied by the density of each species at each site during each sampling period (per m^2^) obtained from density quadrat surveys (g DW m^−2^). The rate of detrital production through dislodgement per day was calculated using a similar construct but was further multiplied by the mean dry biomass of adult kelp individuals and divided by the number of days between sampling (g DW m^−2^). For an annual estimate of production (productivity) and detrital production (erosion and dislodgement) for *L. digitata* and *S. latissima*, seasonally varying rates were averaged over the whole year, and estimates of daily rates were then multiplied by 365 (g DW m^−2^; Krumhansl & Scheibling, 2011).

Factors that may influence growth and detritus production rates, including grazer density and damage, epiphytic algal cover, temperature, and light were also quantified. Temperature and light were measured in situ using HOBO temperature/light Pendant data loggers mounted at each site at the relevant shore height. Detailed methods to quantify these variables and graphs showing annual variation can be found in the Appendix (Figures 3 and 4).

### 2.2 Data analysis

To test for the effects of wave exposure (fixed, two levels), sampling period (fixed, eight levels), and site (random and nested in wave exposure, two levels) on *L. digitata* density, productivity and erosion (individual and per m^−2^), and total detrital production, linear mixed effect models fitted by maximum likelihood were performed using the package *lme*4 (Zuur et al., 2009). Sampling period was treated initially as a fixed factor so that we could test explicitly for putative differences and identify which sampling times differed from each other. All models included an interaction term but when not significant, interactions were removed and the model was re‐fitted with main terms only. If model assumptions were met, type 2 ANOVA was used to obtain *χ* ^2^ and *p*‐values (package *car*; Fox & Weisberg, 2011). Where *p*‐values were significant, Tukey HSD adjusted pairwise comparisons using least‐square means were used for post hoc comparisons (package *lsmeans*; Lenth, 2018). Residuals were visually inspected and QQ plots were used to check assumptions of normality and homogeneity of variance. Where residuals did not meet model assumptions despite the transformation, data were analyzed using a generalized linear mixed model with a Tweedie distribution that also accounts for zero inflation (package *Tweedie*; Arcuti et al., 2013). Where sampling periods contained only one level of wave exposure or site owing to logistical difficulties preventing data collection at certain sites, those time points were excluded from the analysis. Analysis of *S. latissima* followed a similar construct but without wave exposure because this kelp species was only found on the two moderately exposed shores. Owing to only two replicates per treatment, dislodgement rate, and detrital production through dislodgement were not analyzed statistically and only patterns in the data are presented for observation.

To test whether biotic (fixed: distal area grazed, total grazer abundance, epiphytic algal cover) and abiotic (fixed: mean and maximum temperature, mean and maximum light, daily cumulative irradiance, wave exposure) factors affected production and erosion of *L. digitata* and *S. latissima*, linear mixed effect models were used. Site and sampling period were treated as random factors in the model as we were not interested in testing for differences between sampling periods specifically, but for relationships between explanatory and predictor variables. All remaining main terms were included in the model and model selection was performed using Akaike information criterion (AIC) values and weights, where the lowest AIC values represented the optimal model (Aho et al., 2014; Zuur et al., 2009). Residuals were visually inspected and QQ plots were used to check assumptions of normality and homogeneity of variance. Where sampling periods contained only one level of wave exposure or one site owing to logistical difficulties preventing data collection at certain sites, those time points were excluded from the analysis. All analyses were conducted using R version 3.3.4 (R Development Core Team, 2017).

## 3 RESULTS

**FIGURE 2:**
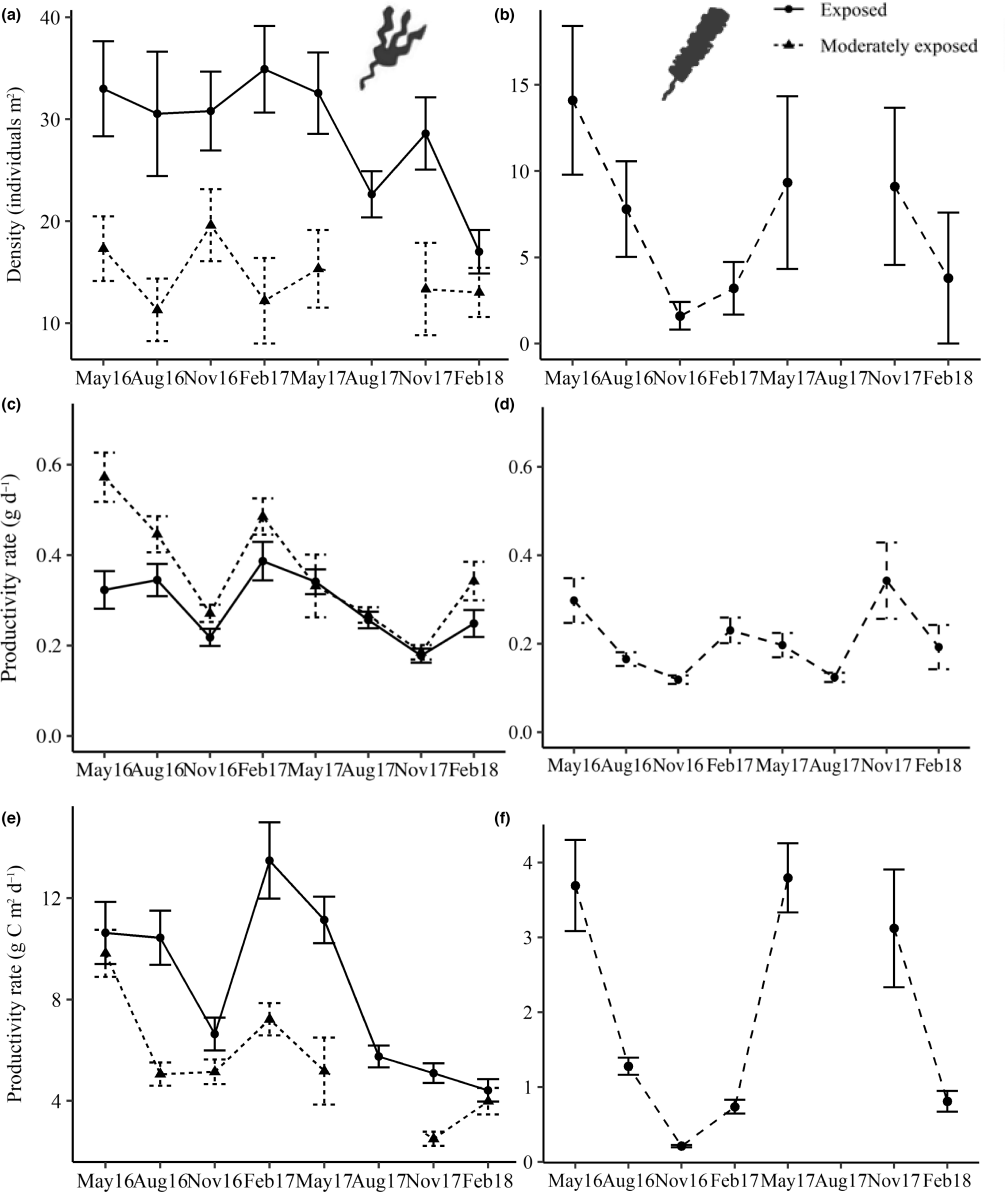
Mean (±SE) density (m^−2^), individual productivity (g DW day^−1^) and productivity per m^−2^ (g DW m^−2^ day^−1^) of *Laminaria digitata* (a, c, and e, respectively) and *Saccharina latissima* (b, d, and f, respectively) based on four sites at two different levels of wave exposure in Co. Donegal, Ireland. *n* = 8–32. Aug, August; Feb, February; Nov, November. Black circles represent sampling periods in which data are unavailable.

The density of *L. digitata* differed among wave exposures ($\chi_{1,2}^{2}$ = 20.484; *p* < .001) and sampling periods ($\chi_{1,6}^{2}$ = 14.175; *p* < .01). Post hoc tests showed that *L. digitata* density on exposed shores (28.74 ± 1.43 individuals per m^2^) was twice that of moderately exposed shores (15.05 ± 1.35 individuals per m^2^) and density was generally greatest in February or May at both exposures during both survey years (Figure 2a). No significant effect of sampling period on the density of *S. latissima* was identified, with density remaining consistently low throughout the survey period ($\chi_{1,6}^{2}$ = 10.28; *p* = .1; 7.32 ± 1.38 individuals per m^2^; Figure 2b).

A significant interaction between wave exposure and sampling period on the productivity of *L. digitata* was identified (Table 1; Figure 2c). Specifically, wave exposures did not differ from each other within sampling periods owing to the variable nature of these data. There were significant differences between sampling periods, however, that were not consistent across wave exposures. Specifically, sampling periods at exposed sites did not differ from each other but at moderately exposed sites, May of 2016 (0.35 ± 0.03 g DW day^−1^) was significantly greater than most other sampling periods and November of 2017 (0.18 ± 0.01 g DW day^−1^) significantly lower (see Table S1a for all post‐hoc comparisons). A significant interaction between wave exposure and sampling period was also identified for productivity per m^2^ of *L. digitata* (Table 1; Figure 2e). As seen for individual productivity, wave exposure levels did not differ within sampling periods but differed between sampling periods inconsistently across wave exposure levels. Specifically, at exposed sites, February of 2017 (13.48 ± 1.5 g DW day^−1^) was greater than most other sampling periods and November of 2017 was significantly lower (2.5 ± 0.28 g DW day^−1^; Table S1b). The productivity of *S. latissima* also differed between sampling periods ($\chi_{1,7}^{2}$ = 25.57; *p* < .001; Figure 2d). Post hoc tests identified the greatest rates in May (0.3 ± 0.05 g DW day^−1^) and lowest in November (0.12 ± 0.01 g DW day^−1^), but conversely, peaked in November in 2017 (0.34 ± 0.08 g DW day^−1^). Productivity per m^2^ of *S. latissima* did not follow patterns of individual productivity rate but rather that of density, with the greatest productivity during May of both 2016 and 2017 ($\chi_{1,6}^{2}$ = 164.37; *p* < .001; Figure 2f).

**TABLE 1:** Linear mixed effects model testing for effects of wave exposure and sampling period on the productivity (g day^−1^), productivity per m^−2^ (g DW day^−2^), and erosion rate (g day^−1^) of *Laminaria digitata*. Samples were collected at four sites, two exposed and two moderately exposed, during eight consecutive sampling periods. Individual sites nested in wave exposure were included as a random factor in the statistical model. Significant results are in bold (*p* < .05).

	Productivity (g day^−1^)			Productivity (g DW m^−2^ day^−1^)		
	df	*χ* ^2^	*p*‐Value	df	*χ* ^2^	*p*‐Value
Wave exposure (W)	1	2.78	.09	1	2.01	.15
Sampling period (SP)	6	72.48	**<.001**	6	76.84	**<.001**
W × SP	6	15.76	**.01**	5	16.64	**.005**

	Erosion (g day^−1^)			Total detrital production (g DW m^−2^ day^−1^)		
	df	*χ* ^2^	*p*‐Value	df	*χ* ^2^	*p*‐Value
Wave exposure (W)	1	5.79	**.01**	1	0.05	.8
Sampling period (SP)	6	97.28	**<.001**	6	73.85	**<.001**
W × SP	6	35.72	**<.001**	5	20.27	**.001**

**FIGURE 3:**
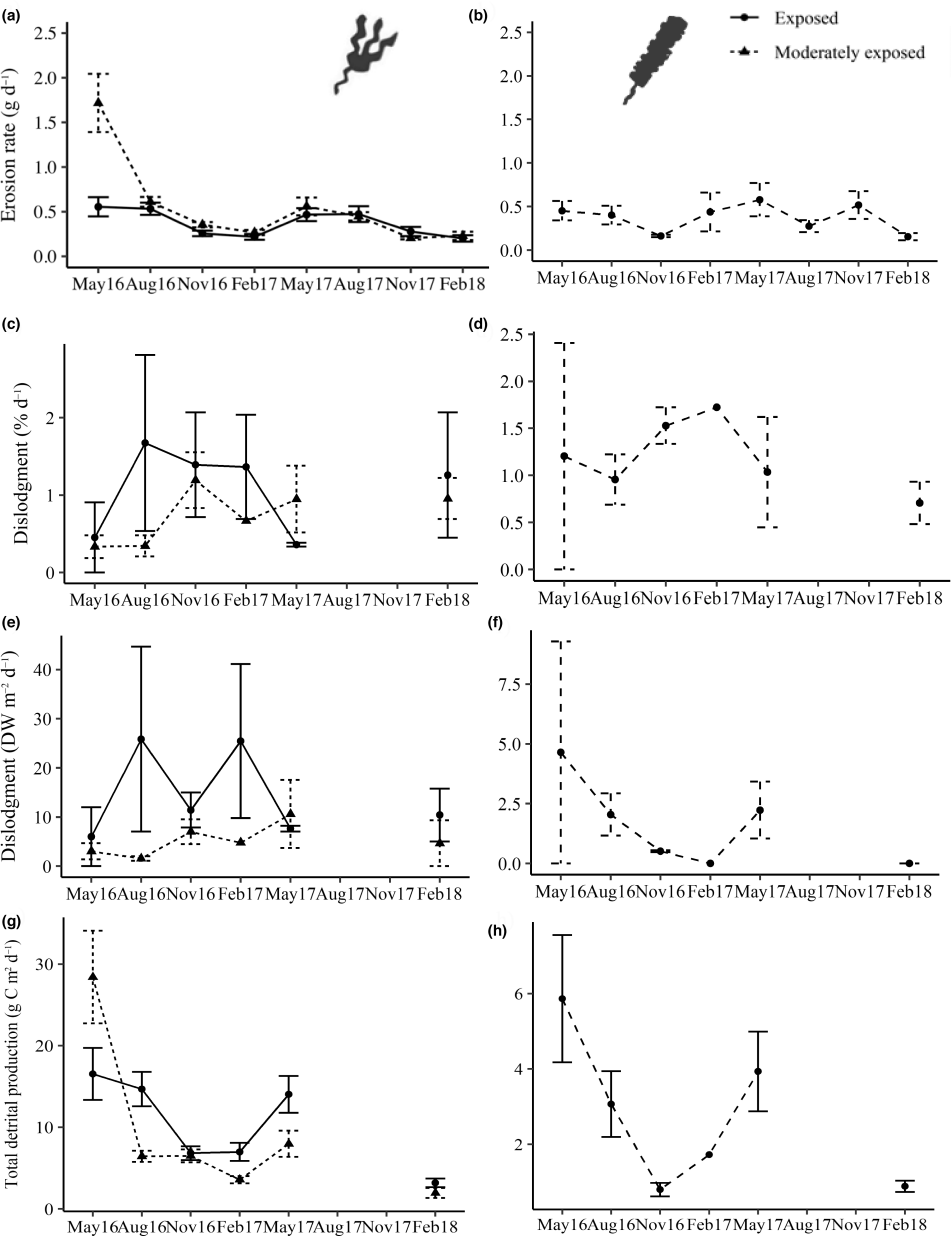
Mean (±SE) rate of erosion (g DW day^−1^), dislodgement (% m^−2^ day^−1^), detrital production through dislodgement (DW m^−2^ day^−1^), and total detrital production (via erosion and dislodgement; g DW m^−2^ day^−1^) of *Laminaria digitata* (a, c, e, and g, respectively) and *Saccharina latissima* (b, d, f, and h, respectively). Data were based on four sites at two different levels of wave exposure in Co. Donegal, Ireland. *n* = 2. Data for August and November, 2017 are unavailable.

Erosion rates did not differ between levels of wave exposure within sampling periods but differed between sampling periods inconsistently across wave exposure levels (Table 1; Figure 3a). Sampling periods at exposed sites did not differ from each other but at moderately exposed sites, May 2016 (1.7 ± 0.32 g DW day^−1^) was significantly greater than all other sampling periods (Table S1c). Erosion rates of *S. latissima* differed between sampling periods ($\chi_{1,7}^{2}$ = 20.43; *p* = .004), with the greatest rates in May in both 2016 and 2017 (0.45 ± 0.11 and 0.57 ± 0.19 g DW day^−1^, respectively) and lowest during November in 2016 but August in 2017 (0.16 ± 0.01 and 0.27 ± 0.06 g DW day^−1^, respectively; Figure 3b).

Although dislodgement data could not be statistically analyzed, it appears that at exposed sites, dislodgement rates of *L. digitata* were greatest in August and February of 2016 and 2017 (Figure 3c). Rates of dislodgement for both *L. digitata* and *S. latissima* at moderately exposed sites, however, were greatest in November 2016 and February 2017 (Figure 3c,d, respectively). Mean detrital production through dislodgement by *L. digitata* was greater at exposed sites during August and February of 2016 and 2017, respectively, but at moderately exposed sites, *L. digitata* and *S. latissima* both peaked in November and May (Figure 3e,f, respectively).

Total detrital production of *L. digitata* did not differ between exposure levels within sampling periods owing to high variability in the dataset but differed inconsistently between sampling periods across levels of wave exposure (Table 1; Figure 3g). At exposed sites, May 2016 (16.52 ± 3.18 g DW day^−1^) was greater than February 2018 only (3.28 ± 0.52 g DW day^−1^). At moderately exposed sites, however, May 2016 (28.4 ± 5.67 g DW day^−1^) was greater than all other sampling periods (Table S1d). Total detrital production of *S. latissima* followed a similar pattern to density (per m^2^) and differed between sampling periods ($\chi_{1,5}^{2}$ = 15.79; *p* = .007), with the greatest detrital production in May of both 2016 and 2017 (Figure 3h).

**FIGURE A2:**
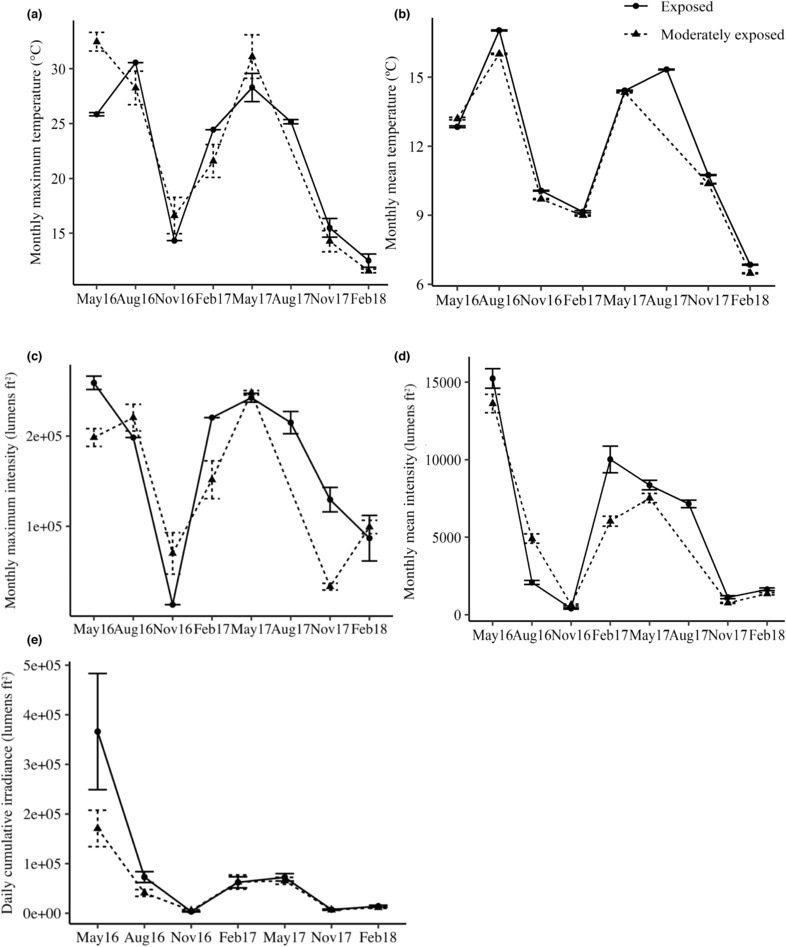
Monthly maximum and mean temperature (a and b, respectively; °C), light intensity (c and d, respectively; lumens ft^2^), and daily cumulative irradiance (e; lumens ft^2^). Data were based on two sites at two different levels of wave exposure (exposed and moderately exposed; *n* = 2 per level of wave exposure) in Co. Donegal, Ireland.

**FIGURE A3:**
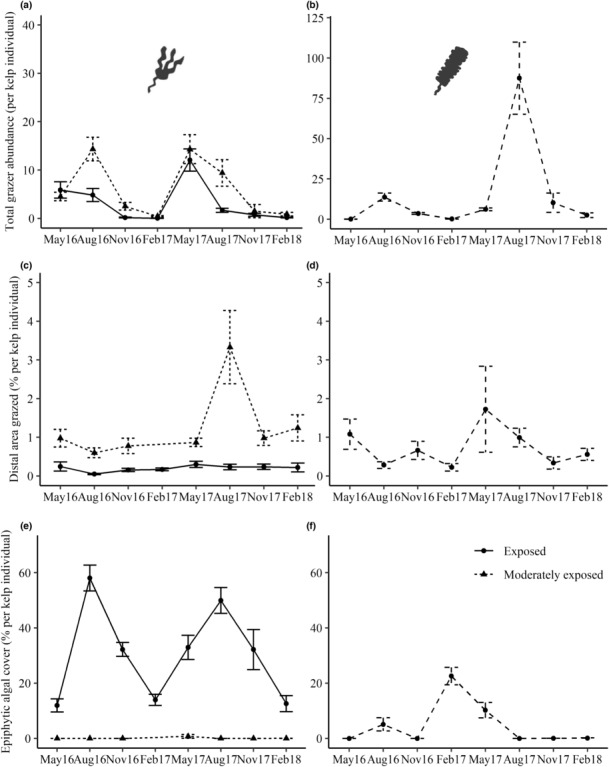
Mean (±1 SE) total grazer abundance (per kelp individual), distal area grazed (% per kelp individual), and epiphytic algal cover (per individual) of *Laminaria digitata* (a, c, and e, respectively) and *Saccharina latissima* (b, d, and f, respectively). Data were based on two sites at two different levels of wave exposure (exposed and moderately exposed; *n* = 2 per level of wave exposure) in Co. Donegal, Ireland. *n* = 10–37.

Monthly mean (negatively related) and maximum (positively related) temperature were identified as key factors affecting individual productivity rates of *L. digitata* (*R* ^2^ = 33.8%; Table 2). Similarly, individual productivity rates of *S. latissima* were correlated with monthly mean (negatively related) and maximum (positively related) temperature and maximum light (negatively related; *R* ^2^ = 26.4%; Table 2). Wave exposure (positively related), monthly mean (positively related), maximum (negatively related) temperature, maximum light (negatively related), daily cumulative irradiance (negatively related), grazing (positively related), and epiphyte cover (positively related) were identified as factors affecting the individual erosion rate of *L. digitata* (*R* ^2^ = 31.8%; Table 2). Individual erosion rates of *S. latissima*, however, were correlated with monthly mean (positively related) and maximum temperature (negatively related; *R* ^2^ = 16.4%; Table 2; Figures illustrating all quantified variables are in Figures A2 and A3).

**TABLE 2:** The best models of abiotic (wave exposure [WE], maximum [*T*_max_] and mean monthly temperature [*T*_avg_]), maximum monthly light (*L*_max_), daily cumulative irradiance (DCI), and biotic (epiphytic algal cover [*E*%], distal area grazed [*G*%], and total grazer abundance [Abun]) factors identified to explain variation in productivity (g day^−1^) and erosion (g day^−1^) for *Laminaria digitata* and *Saccharina latissima*.

Variable	Intercept	Model parameters + slope	Weight	*R* ^2^
*L. digitata*				
Productivity	−1.757	*T*_max_ (0.015), *T*_avg_ (−0.029)	0.162	.338
Erosion	−4.956	WE (+), *T*_max_ (−0.022), *T*_avg_ (27.14), *L*_max_ (−0.0002), DCI (−0.00002), *E*% (16.65), *G*% (46.90)	0.902	.318
*S. latissima*				
Productivity	−1.144	*T*_max_ (0.089), *T*_avg_ (−0.126) *L*_max_ (−0.0007)	0.162	.264
Erosion	−6.996	*T*_max_ (−0.6946), *T*_avg_ (3.192)	0.121	.194

## 4 DISCUSSION

We identified a seasonal pattern in individual productivity rates for *L. digitata* and *S. latissima* that is aligned with many other kelp species globally, with a peak in production in late winter and spring (February/May) and seasonal low in autumn (November; Brady‐Champbell et al., 1984; Fairhead & Cheshire, 2004; Krumhansl & Scheibling, 2011; Mann, 1973; Miller et al., 2011; Pessarrodona, Moore, et al., 2018; Tala & Edding, 2005). This cycle is driven by changes in photoperiod and annual temperature fluctuations, which are in turn linked to nutrient dynamics and wave exposure (Bekkby et al., 2014; Hepburn et al., 2007; Kain, 1979; Pedersen et al., 2012; Reed et al., 2011). This is supported by the identification of temperature and light as key factors affecting individual productivity rates of these kelp species, accounting for between 26% and 34% of the observed variation in the data. Peak growth rates of *L. digitata* (0.39–0.49 g DW day^−1^) and *S. latissima* (0.34 DW g day^−1^) were lower than estimates for their subtidal counterpart *L. hyperborea* (0.78–0.87 g DW day^−1^) and the warm‐water kelp *Laminaria ochroleuca* (0.63 g DW day^−1^; Pessarrodona, Foggo, et al., 2018). On an annual basis, however, owing to their continual growth throughout the year, mean annual productivity rates are comparable across species throughout the region (*L. digitata* 0.29–0.38 g DW day^−1^; *L. hyperborea* 0.19 g DW day^−1^; *L. ochroleuca* 0.33–0.37 g DW day^−1^; Pessarrodona, Foggo, et al., 2018). Predicted increases in temperature under climate change scenarios (IPCC, 2022) are, therefore, likely to significantly reduce the productivity of these kelp species, slowing rates of carbon fixation and storage (Harley et al., 2006; Pessarrodona, Moore, et al., 2018).

Both studied species released detritus via erosion of the distal parts of the blade throughout the year, providing a consistent flow of organic matter from kelp stands. This is in contrast to another co‐occurring species *L. hyperborea* which is characterized by a discrete phase of detrital production in which the old lamina is shed during the months of March–May (Kain & Jones, 1971; Pessarrodona, Foggo, et al., 2018; Pessarrodona, Moore, et al., 2018). Peak erosion rates of *L. digitata* at both wave exposures ranged between 0.6 and 1.7 g DW day^−1^ and were ~0.6 g DW day^−1^ for *S. latissima*, which is higher than previous rates recorded for populations of *L. hyperborea* and *L. ochroleuca* along the UK coastline (Pessarrodona, Moore, et al., 2018). Seasonal lows for both *L. digitata* (0.2–0.26 g DW day^−1^) and *S. latissima* (0.26 g DW day^−1^) were still greater than the mean annual erosion rate of *L. hyperborea* (~0.19 g DW day^−1^) and only marginally lower than *L. ochroleuca* (~0.33 g DW day^−1^; Pessarrodona, Foggo, et al., 2018). When considering habitat extent, however, it is likely that *L. hyperborea* populations make greater contributions to the detritus pool, given the greater areal coverage and depth penetration than *L. digitata* (Smith et al., 2022). Even so, the contribution of intertidal kelp stands to coastal detrital pools, which has been largely overlooked, is likely to be significant.

Wave exposure was identified as a significant factor positively affecting erosion rates of *L. digitata*, which is in line with previous studies in other regions (de Bettignies et al., 2012, 2013; Krumhansl & Scheibling, 2011). In intertidal habitats, individuals are subjected to heavy wave action that can cause physical damage (i.e., abrasion, breakage) and contribute to detrital production (Dobrynin et al., 2010; Mach et al., 2007). Erosion rates of *L. digitata* and *S. latissima* were also correlated with temperature, light, grazing, and epiphytic algal cover, all of which fluctuated markedly throughout the survey period and exhibit high seasonality (Figures A2 and A3). Increased temperature has been linked to tissue degradation in kelps, reducing tensile strength and increasing susceptibility to erosion during warm periods (Krumhansl & Scheibling, 2011, 2012; Rothäusler et al., 2009). Higher temperatures experienced during summer are, however, also associated with increased grazer abundances and consumption rates that can further exacerbate tissue damage (Gilson, Smale, & O'Connor, 2021; Krumhansl & Scheibling, 2011; Toth & Pavia, 2002). Increased cover of epiphytes also generally occurs through summer when temperatures are high, is often indicative of senescing kelp tissue, and can increase breakage and detritus production (Scheibling & Gagnon, 2009). While it is not possible to disentangle the relative importance of these factors in the current study, particularly when variability is high, it is likely they influenced detrital production rates and may to some extent explain the observed variability between survey years. It is also likely that other factors not considered in this study are important drivers of detritus production, in particular for *S. latissima* in which only a small proportion of the observed variation was explained by the predictor variables included in the model. For example, the production of reproductive sorus tissue in kelps, which also varies seasonally, has previously been linked to detrital production rates and may have accounted for increased erosion throughout autumn and winter (de Bettignies et al., 2013).

Although data for dislodgement was not statistically analyzed and variability was high, there is some tentative evidence of differences based on shore and sampling period. Dislodgement rates and detrital production through dislodgement of *L. digitata* were greater at exposed sites, and during August–February at both levels of wave exposure, which coincides with increased dislodgement during periods of heavy wave action. August to November is hurricane season in the NW Atlantic, bringing strong westerly winds and large swells across the Atlantic, while December–February is the winter period in NE Atlantic (e.g., Brown et al., 2010; Wolf & Woolf, 2006). Although individuals of *L. digitata* were larger on average than *S. latissima* and contributed greater quantities of detritus to the detrital pool, the ruffled margins of *S. latissima* create considerably more drag than the flat lamina of *L. digitata*, accounting for their greater rates of dislodgment even at more sheltered sites (Buck & Buchholz, 2005). *S. latissima* also routinely settles on semi‐stable rocks and cobbles instead of emergent bedrock, particularly in sheltered conditions, increasing their susceptibility to dislodgement (Scheibling et al., 2009; Smale & Vance, 2016). In addition, individuals of *L. digitata* are morphologically adapted to wave‐exposed conditions, with a larger, stronger holdfast and stipe and more streamlined blades that enable greater attachment to the substrata and reduce drag. Although dislodgement rates for both kelp species were lower than those reported for subtidal *L. hyperborea* populations (4%–27% m^−2^ year^−1^; Pessarrodona, Moore, et al., 2018; Smale et al., 2022), most likely because of the degree of protection subtidal kelp forests offer intertidal kelp beds, the greater population densities of *L. digitata* in intertidal habitats recorded here resulted in a much larger contribution to the detrital pool per unit area. Clearly, predicted increases in storm frequency are likely to lead to greater rates of dislodgement (Feser et al., 2015; IPCC, 2022), potentially increasing detrital resources within coastal food webs.

**FIGURE 4:**
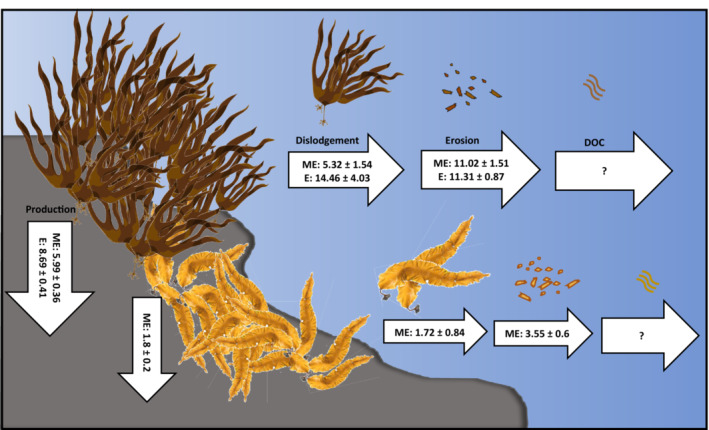
Schematic showing the mean (±SE) amount of carbon (g DW m^−2^ day^−1^) fixed through primary production and lost through detrital production (dislodgement, erosion, and dissolved organic carbon (DOC) annually for *Laminaria digitata* and *Saccharina latissima* at two moderately exposed (ME) and two exposed (*E*) Irish shores. *n* = 15–168.

Overall, erosion (rather than dislodgement) was the dominant mechanism of detrital production for both *L. digitata*, at exposed and moderately exposed sites, and *S. latissima*, accounting for 72%, 77%, and 77% of total detrital production, respectively (Figure 4). Total detrital production was greatest at exposed sites for *L. digitata* (25.77 g DW m^−2^ day^−1^) owing to greater rates of dislodgement. Scaled annually, *L. digitata* produces 9.4 kg DW m^−2^ year^−1^ of detritus on exposed and 5.96 kg DW m^−2^ year^−1^ on moderately exposed shores and *S. latissima* produces 1.9 kg DW m^−2^ year^−1^ of detritus on moderately exposed shores. Although we did not measure detrital production during every month of the year and may have missed smaller‐scale patterns associated with storms, we have captured the seasonal dynamics and larger‐scale patterns of these processes. However, the lack of reliable spatial extent data for either species, particularly within intertidal and shallow subtidal habitats in the UK and Ireland, makes scaling‐up to whole coastlines and seascapes challenging. Even so, the total contribution of intertidal kelp stands to local and regional detrital pools and coastal carbon cycles is likely to be significant. A major knowledge gap relates to the ultimate fate of this detrital material, in terms of how quickly it is consumed and remineralized, whether it subsidizes receiver habitats, and whether any kelp‐derived carbon is stored in sink habitats for meaningful timescales.

In conclusion, we have shown that intertidal kelp beds constitute a significant carbon flux and are major contributors to coastal productivity and detritus production, highlighting the need for these habitats to be incorporated into ecosystem models. Previous estimations of macroalgal contributions to coastal carbon cycles have generally focused on intertidal fucoids and subtidal populations of kelp (Pessarrodona et al., 2022). It is important to note, however, that the rate estimates presented here were obtained from a limited number of sites within a region where such information is very scarce (Schoenrock et al., 2020, 2021). For *L. digitata*, population densities were at the higher end of previous estimates, and individual size far exceeded previously reported values, resulting in very high estimates of productivity and detrital production. In addition, *S. latissima* typically dominates sheltered shorelines that were not the focus of the current study, so that the contribution of this species to regional carbon budgets and food webs is probably even greater than suggested here. Further mensurative studies are needed across greater spatial scales, to incorporate multiple *L. digitata* and *S. latissima* populations and a wider range of environmental conditions. Improving our knowledge of the role these habitats play in coastal and global cycles is critical to understanding climate‐driven change and implementing management plans with a climate‐change mitigation perspective (Duarte, 2017).

## AUTHOR CONTRIBUTIONS

**Abby R. Gilson:** Conceptualization (lead); data curation (lead); formal analysis (lead); investigation (lead); methodology (lead); visualization (lead); writing – original draft (lead); writing – review and editing (lead). **Dan A. Smale:** Conceptualization (supporting); formal analysis (supporting); methodology (supporting); supervision (supporting); visualization (supporting); writing – review and editing (supporting). **Michael T. Burrows:** Conceptualization (supporting); formal analysis (supporting); methodology (supporting); supervision (supporting); visualization (supporting); writing – review and editing (supporting). **Lydia J. White:** Formal analysis (supporting); investigation (supporting); methodology (supporting); visualization (supporting); writing – review and editing (supporting). **Nessa E. O'Connor:** Conceptualization (supporting); formal analysis (supporting); funding acquisition (lead); methodology (supporting); supervision (lead); visualization (supporting); writing – review and editing (supporting).

## CONFLICT OF INTEREST STATEMENT

The authors have no conflict of interest to declare.

## Supporting information


Table S1.

## Data Availability

All data is available from the British Oceanographic Data Centre. DOI: https://doi.org/10.5285/bb7366a9‐e053‐6c86abc0cea1.

## METHODS TO QUANTIFY POTENTIAL ABIOTIC AND BIOTIC FACTORS AFFECTING KELP PRODUCTION AND DETRITAL PRODUCTION

### 1

To identify the biotic factors that may affect kelp production and breakdown and to test whether these effects differed among dominant kelp species, 15 individuals of *Laminaria digitata* and *Saccharina latissima* at each site were surveyed for the presence of grazers, with grazer identity and density per kelp individual recorded. The distal part of the blades of each individual kelp was then placed between two sheets of plexiglass and photographed. Only the distal 1/3rd of the kelp individual was measured because erosion is known to occur primarily in the distal portion of the blade and grazing and epiphytic algal cover were observed to be concentrated in this region (Krumhansl & Scheibling, 2011). Distal area grazed (estimated using perforations of the blade only) and percentage epiphytic algal cover were then calculated by dividing the total area by the area grazed and the area covered by epiphytic algae, respectively, using ImageJ.

To identify abiotic factors that may affect kelp production and breakdown, temperature (°C) and light (lumens ft^2^) were recorded every 15 min and averaged for each site during each sampling period using data loggers (*n* = 8; HOBO temperature/light weatherproof Pendant data Logger 16k, Onset). Mean and monthly temperature and light and daily cumulative irradiance were then calculated for each site during each sampling period. As loggers were placed intertidally, estimates include periods of low tide emersion and therefore air temperature. Data were not available for sampling period 6 (August 2017) owing to adverse weather conditions preventing the collection or loss of the loggers.
