# Supplementary material for: Seasonal and spatial variability in rates of primary production and detritus release by intertidal stands of Laminaria digitata and Saccharina latissima on wave‐exposed shores in the northeast Atlantic
Source: Ecol Evol. 2023 Jun 20;13(6):e10146. doi: 10.1002/ece3.10146 (PMC10282169; doi:10.1002/ece3.10146)
Supplement: Supplementary file 1 — Table S1. [file ECE3-13-e10146-s001.docx]

Table S1. Post-hoc comparisons (Lsmeans) on the effects of wave exposure (exposed and moderately exposed) and sampling period on a) individual productivity rate (g d^-1^), b) productivity per m^2^ (g C m^2^ d^-2^), c) individual erosion rate (g d^-1^) and d) total detrital production (g C m^2^ d^-1^).

| *a) Post-hoc* test | *p-*value |
| --- | --- |
| Exposed |  |
| May16-Aug16  May16-Nov16  May16-Feb17  May16-May17  May16-Nov17  May16-Feb18  Aug16-Nov16  Aug16-Feb17  Aug16-May17  Aug16-Nov17  Aug16-Feb18  Nov16-Feb17  Nov16-May17  Nov16-Nov17  Nov16-Feb18  Feb17-May17  Feb17-Nov17  Feb17-Feb18  May17-Nov17  May17-Feb18  Nov17-Feb18 | 1  0.9  0.99  1  0.6  0.99  0.81  1  1  0.47  0.99  0.28  0.66  1  1  0.99  0.1  0.99  0.33  0.97  0.99 |
| Moderately exposed |  |
| May16-Aug16  May16-Nov16  May16-Feb17  May16-May17  May16-Nov17  May16-Feb18  Aug16-Nov16  Aug16-Feb17  Aug16-May17  Aug16-Nov17  Aug16-Feb18  Nov16-Feb17  Nov16-May17  Nov16-Nov17  Nov16-Feb18  Feb17-May17  Feb17-Nov17  Feb17-Feb18  May17-Nov17  May17-Feb18  Nov17-Feb18 | 0.39  **0.03**  0.46  **< 0.001**  **0.01**  **0.001**  **0.02**  1  0.6  0.15  0.7  **0.003**  0.99  0.92  0.98  0.24  **< 0.001**  0.31  0.4  1  0.26 |

| *b) Post-hoc* test | *p-*value |
| --- | --- |
| Exposed |  |
| May16-Aug16  May16-Nov16  May16-Feb17  May16-May17  May16-Nov17  May16-Feb18  Aug16-Nov16  Aug16-Feb17  Aug16-May17  Aug16-Nov17  Aug16-Feb18  Nov16-Feb17  Nov16-May17  Nov16-Nov17  Nov16-Feb18  Feb17-May17  Feb17-Nov17  Feb17-Feb18  May17-Nov17  May17-Feb18  Nov17-Feb18 | 1  0.16  0.63  1  **0.01**  **0.004**  0.36  0.6  1  **0.04**  **0.02**  **< 0.001**  **0.03**  0.99  0.97  0.84  **< 0.001**  **< 0.001**  **0.002**  **0.004**  1 |
| Moderately exposed |  |
| May16-Aug16  May16-Nov16  May16-Feb17  May16-May17  May16-Nov17  May16-Feb18  Aug16-Nov16  Aug16-Feb17  Aug16-May17  Aug16-Nov17  Aug16-Feb18  Nov16-Feb17  Nov16-May17  Nov16-Nov17  Nov16-Feb18  Feb17-May17  Feb17-Nov17  Feb17-Feb18  May17-Nov17  May17-Feb18  Nov17-Feb18 | **0.002**  **0.003**  0.91  **0.01**  **0.001**  **0.03**  1  0.97  1  0.95  1  0.97  1  0.92  1  0.98  0.27  0.94  0.94  1  1 |

| *c) Post-hoc* test | *p-*value |
| --- | --- |
| Exposed |  |
| May16-Aug16  May16-Nov16  May16-Feb17  May16-May17  May16-Nov17  May16-Feb18  Aug16-Nov16  Aug16-Feb17  Aug16-May17  Aug16-Nov17  Aug16-Feb18  Nov16-Feb17  Nov16-May17  Nov16-Nov17  Nov16-Feb18  Feb17-May17  Feb17-Nov17  Feb17-Feb18  May17-Nov17  May17-Feb18  Nov17-Feb18 | 1  0.93  0.86  1  0.98  0.86  0.95  0.88  1  0.98  0.89  1  0.99  1  1  0.98  1  1  0.99  0.98  1 |
| Moderately exposed |  |
| May16-Aug16  May16-Nov16  May16-Feb17  May16-May17  May16-Nov17  May16-Feb18  Aug16-Nov16  Aug16-Feb17  Aug16-May17  Aug16-Nov17  Aug16-Feb18  Nov16-Feb17  Nov16-May17  Nov16-Nov17  Nov16-Feb18  Feb17-May17  Feb17-Nov17  Feb17-Feb18  May17-Nov17  May17-Feb18  Nov17-Feb18 | **< 0.001**  **< 0.001**  **< 0.001**  **< 0.001**  **< 0.001**  **< 0.001**  0.94  0.73  1  0.74  0.59  1  0.99  1  1  0.93  1  1  0.91  1  1 |

| *d) Post-hoc* test | *p-*value |
| --- | --- |
| Exposed |  |
| May16-Aug16  May16-Nov16  May16-Feb17  May16-May17  May16-Feb18  Aug16-Nov16  Aug16-Feb17  Aug16-May17  Aug16-Feb18  Nov16-Feb17  Nov16-May17  Nov16-Feb18  Feb17-May17  Feb17-Feb18  May17-Feb18 | 1  0.29  0.31  0.99  **0.04**  0.57  0.59  1  0.15  1  0.73  0.99  0.77  0.99  0.19 |
| Moderately exposed |  |
| May16-Aug16  May16-Nov16  May16-Feb17  May16-May17  May16-Feb18  Aug16-Nov16  Aug16-Feb17  Aug16-May17  Aug16-Feb18  Nov16-Feb17  Nov16-May17  Nov16-Feb18  Feb17-May17  Feb17-Feb18  May17-Feb18 | **< 0.001**  **< 0.001**  **< 0.001**  **< 0.001**  **< 0.001**  1  1  1  0.94  1  1  0.92  1  0.99  0.81 |
